# RETRACTED: Maiti et al. Ameliorative Properties of Boronic Compounds in In Vitro and In Vivo Models of Alzheimer’s Disease. *Int. J. Mol. Sci.* 2020, *21*, 6664

**DOI:** 10.3390/ijms26136396

**Published:** 2025-07-03

**Authors:** Panchanan Maiti, Jayeeta Manna, Zoe N. Burch, Denise B. Flaherty, Joseph D. Larkin, Gary L. Dunbar

**Affiliations:** 1 Field Neurosciences Institute Laboratory for Restorative Neurology, Central Michigan University, Mt. Pleasant, MI 48859, USA; 2 Department of Psychology, Central Michigan University, Mt. Pleasant, MI 48859, USA; 3 Program in Neuroscience, Central Michigan University, Mt. Pleasant, MI 48859, USA; 4 Field Neurosciences Institute, Ascension St. Mary, Saginaw, MI 48604, USA; jayeeta.manna@ascension.org; 5 College of Health and Human Services, Saginaw Valley State University, Saginaw, MI 48604, USA; 6 Department of Biology, Eckerd College, St. Petersburg, FL 33711, USA; znburch@eckerd.edu (Z.N.B.); flaherdb@eckerd.edu (D.B.F.); 7 Department of Chemistry, Eckerd College, St. Petersburg, FL 33711, USA; larkinjd@eckerd.edu

The journal retracts the article titled “Ameliorative Properties of Boronic Compounds in In Vitro and In Vivo Models of Alzheimer’s Disease” [1], cited above.

Following publication, concerns were brought to the attention of the Editorial Office regarding a range of image irregularities contained within this article [1].

Adhering to our complaints procedure, the Editorial Office and Editorial Board conducted an investigation that confirmed a partial overlap of sub-images in Figures 4A, 5A, and 6A showing different experimental conditions, as well as the overlap between a sub-image in Figure 3A in publication [1] and a sub-image in Figure 3 from a different publication [2]. While the authors fully cooperated with the Editorial Office, they were not able to satisfactorily explain the presence of the above-mentioned irregularities. Consequently, the Editorial Board and the authors decided to retract this publication [1], as per MDPI’s retraction policy (https://www.mdpi.com/ethics#_bookmark30).

This retraction was approved by the Editor-in-Chief of the *International Journal of Molecular Sciences* journal.

Gary L. Dunbar, Denise B. Flaherty and Joseph D. Larkin agreed to this retraction. The remaining authors did not provide a comment on this decision.

## References

[1] Maiti P., Manna J., Burch Z.N., Flaherty D.B., Larkin J.D., Dunbar G.L. (2020). RETRACTED: Ameliorative Properties of Boronic Compounds in In Vitro and In Vivo Models of Alzheimer’s Disease. Int. J. Mol. Sci..

[2] Maiti P., Bowers Z., Bourcier-Schultz A., Morse J., Dunbar G.L. (2021). Preservation of dendritic spine morphology and postsynaptic signaling markers after treatment with solid lipid curcumin particles in the 5xFAD mouse model of Alzheimer’s amyloidosis. Alzheimers Res. Ther..

